# Supplementary material for: Ab Initio Calculations of Quantum Light–Matter Interactions in General Electromagnetic Environments
Source: J Chem Theory Comput. 2024 Jan 8;20(2):926–36. doi: 10.1021/acs.jctc.3c00967 (PMC10809713; doi:10.1021/acs.jctc.3c00967)
Supplement: Supplementary file 1 — ct3c00967_si_001.pdf [file ct3c00967_si_001.pdf]

Supplementary Information: Ab initio calculations of quantum light-matter interactions in general electromagnetic environments

Mark Kamper Svendsen,[*,†,‡,¶] Kristian Sommer Thygesen,[‡] Angel Rubio,[†,¶,§] and Johannes Flick[‖,¶,⊥]

†*Max Planck Institute for the Structure and Dynamics of Matter and Center for Free-Electron Laser Science & Department of Physics, Luruper Chaussee 149, 22761 Hamburg, Germany*

‡*Computational Atomic scale Materials Design (CAMD), Department of Physics, Technical University of Denmark, 2800 Kgs. Lyngby, Denmark*

¶*Center for Computational Quantum Physics, Flatiron Institute, New York, New York 10010, USA*

§*Nano-Bio Spectroscopy Group and European Theoretical Spectroscopy Facility (ETSF), Universidad del País Vasco (UPV/EHU), Av. Tolosa 72, 20018 San Sebastian, Spain*

‖*Department of Physics, City College of New York, New York, New York 10031, USA*

⊥*Department of Physics, The Graduate Center, City University of New York, New York, New York 10016, USA*

E-mail: mark-kamper.svendsen@mpsd.mpg.de

Supplementary Note A: Length-gauge Pauli Fierz Hamiltonian in terms of the emitter centered modes

Our starting point is the PZW gauge Hamiltonian describing an electronic system coupled to the MQED field within the dipole approximation.[1] Neglecting magnetic interactions, the Hamiltonian reads,

$$\begin{aligned}\mathcal{H} =& \sum_{\alpha} \frac{\hat{\boldsymbol{p}}_{\alpha}^{2}}{2m_{\alpha}} + \frac{1}{2\epsilon_0} \int d\boldsymbol{r} \hat{\boldsymbol{P}}(\boldsymbol{r})^2 \\ &+ \sum_{\lambda} \int_0^{\infty} d\omega \hbar\omega \int d\boldsymbol{r} \hat{\boldsymbol{f}}_{\lambda}^{\dagger}(\boldsymbol{r},\omega) \cdot \hat{\boldsymbol{f}}_{\lambda}(\boldsymbol{r},\omega) - \hat{\boldsymbol{d}} \cdot \hat{\boldsymbol{E}}(\boldsymbol{r}_0). \end{aligned} \tag{1}$$

To connect this to the Hamiltonian used in the Octopus code QEDFT implementation,[2]

$$\hat{H}_0 = \hat{H}_e + \frac{1}{2} \sum_{\alpha=1}^{N} \left(\hat{p}_{\alpha}^2 + \omega_{\alpha}^2 \left[\hat{q}_{\alpha} + \frac{\boldsymbol{\lambda}_{\alpha}}{\omega_{\alpha}} \cdot \hat{\boldsymbol{R}} \right]^2 \right), \tag{2}$$

we follow Ref.[3] in defining the cavity field strength, $\boldsymbol{\lambda}_i(\omega) = \left(\frac{2}{\hbar\omega}\right)^{1/2} e\boldsymbol{E}_i(\boldsymbol{r}_0, \omega)$, and rewrite the emitter-centered representation of the MQED field in Eq. 6 from the manuscript as,

$$\hat{\boldsymbol{E}}(\boldsymbol{r}_0) = \sum_i \int_0^{\infty} d\omega \left(\frac{\hbar\omega}{2e^2} \right)^{1/2} \boldsymbol{\lambda}_i(\omega) \hat{C}_i(\omega) + \text{h.c.}. \tag{3}$$

Next we want to change from the ladder operators to the canonical operators, $\hat{q}_i(\omega) = \left(\frac{\hbar}{2\omega}\right)^{1/2} (\hat{C}_i(\omega) + \hat{C}_i^{\dagger}(\omega))$ and $\hat{p}_i(\omega) = \left(\frac{\hbar\omega}{2}\right)^{1/2} (\hat{C}_i(\omega) - \hat{C}_i^{\dagger}(\omega))$, again to be consistent with the Octopus implementation of QEDFT. This is possible as long as the orthogonalization scheme used in the construction of $\hat{C}_i(\omega)$ results in real valued $\boldsymbol{V}(\omega)$ matrices. With this change, the electric field operator becomes,

$$\hat{\boldsymbol{E}}(\boldsymbol{r}_0) = \sum_i \int_0^{\infty} d\omega \frac{\omega}{e} \boldsymbol{\lambda}_i(\omega) \hat{q}_i(\omega). \tag{4}$$

The idea is then to use Eq. 4 to put Eq. 1 in a form similar to the Hamiltonian in Eq. 2.

The intuitive generalization of the Hamiltonian to the continuous case suggests that,

$$\begin{aligned}\mathcal{H} =& \mathcal{H}_{\text{Mat}}^{\text{C}} + \\ & \frac{1}{2}\sum_i \int_0^\infty d\omega \left\{ \hat{p}_i(\omega)^2 + \omega^2 \left[\hat{q}_i(\omega) + \frac{\boldsymbol{\lambda}_i(\omega)}{\omega} \cdot \hat{\boldsymbol{R}} \right]^2 \right\}.\end{aligned} \tag{5}$$

Here $\hat{\boldsymbol{R}} = \sum_i \hat{\boldsymbol{r}}_i$ is the center of mass position of the charges making up the emitter which is related to the dipole moment as $\hat{\boldsymbol{d}} = -e\hat{\boldsymbol{R}}$. The question is then under what conditions this holds. Inserting the field expansion into the emitter-field coupling term in the Hamiltonian results in,

$$\hat{H}_{\text{int}} = -\hat{\boldsymbol{d}} \cdot \hat{\boldsymbol{E}}(\boldsymbol{r}_0) = \sum_i \int_0^\infty d\omega\omega \left[\boldsymbol{\lambda}_i(\omega) \cdot \hat{\boldsymbol{R}} \right] \hat{q}_i(\omega). \tag{6}$$

which is the straightforward generalization of the lossless case.

The other term in which the field parameters appear is the dipole self energy term. This term should generally be derived in terms of the transverse projection of the full polarization term,[4]

$$\hat{\boldsymbol{P}}_\perp(\boldsymbol{r}) = \frac{2}{3}\hat{\boldsymbol{P}}(\boldsymbol{r}) + \int d\boldsymbol{r'}\, \hat{\boldsymbol{T}}_\perp(\boldsymbol{r}, \boldsymbol{r'}) \cdot \hat{\boldsymbol{P}}(\boldsymbol{r'}). \tag{7}$$

However, the direct application of this projection is computationally cumbersome. In the lossless case, the Helmholtz equation is Hermitian and its transverse solutions form a orthogonal and complete basis for the transverse space. Consequently, the transverse polarization can be expanded in terms of these modes and the projection can be simplified significantly. This procedure leads to the standard form of the dipole self-energy term,

$$\frac{1}{2\epsilon_0} \int d\boldsymbol{r} \left[\hat{\boldsymbol{P}}_\perp(\boldsymbol{r}) \right]^2 = \frac{1}{2} \sum_\alpha \left[\boldsymbol{\lambda}_\alpha \cdot \hat{\boldsymbol{R}} \right]^2. \tag{8}$$

The fact that the transverse modes can be used to span the transverse projector indicates that

any truncation of the photon Hilbert space needs to be performed carefully as it indirectly results in a truncation of the transverse basis and therefore also the transverse polarization. The two should therefore generally always be truncated consistently.[5]

The expansion of the transverse polarization field in the presence of losses is more complicated. If the cavity losses are not true absorptive losses, but simply a result of e.g. finite reflectivity of the cavity mirrors, the modes will globally obey,

$$\int d\boldsymbol{r} \boldsymbol{E}_i(\boldsymbol{r}, \omega_1) \cdot \boldsymbol{E}_j(\boldsymbol{r}, \omega_2) = \delta_{ij}\delta(\omega_1 - \omega_2), \tag{9}$$

and thus remain orthogonal and complete. In this case the transverse polarization can be straightforwardly generalized,

$$\frac{1}{2\epsilon_0} \int d\boldsymbol{r} \left[\hat{\boldsymbol{P}}_\perp(\boldsymbol{r})\right]^2 = \frac{1}{2} \sum_i \int_0^\infty d\omega \left[\boldsymbol{\lambda}_i(\omega) \cdot \boldsymbol{R}\right]^2. \tag{10}$$

In the presence of true absorption losses it is not possible to define the transverse projector in terms of the field modes because these no longer form a complete, orthonormal set. We speculate that the transverse projection should instead by defined in terms of a biorthonormal construction as discussed in Ref.[6] From the point of the emitter it should not be possible to distinguish these two cases because it only samples the field at its position. We thus speculate that, at least for a single emitter position, the true form of the dipole self energy term in the presence of losses will functionally be no different than Eq. 10. For multiple emitter positions it is unlikely that the dipole self-energy term can be expanded directly in terms of the mode functions, and we will explore this further in future research.

Supplementary Note B: Dyadic Green's Function of the spherical microcavity

We consider the spherically layered microcavity from Ref.[7] To use the emitter-centered framework, the DGF must be derived. In general, the source and field points ($\boldsymbol{r}$ and $\boldsymbol{r'}$ respectively) can be in either the same layer or different layers. Consequently, the DGF for the reflected field is a 9-component object, where each component is a 3D dyad. Labeling the possible combinations of source and field points by two extra indices $m, n = 1, 2, 3$ and invoking the superposition principle stating that the total field will be the superposition of the free dipole field and the reflected field,[8,9] the full DGF of the system can be written as,

$$\boldsymbol{G}_{mn}(\boldsymbol{r}, \boldsymbol{r'}, \omega) = \boldsymbol{G}^{\mathrm{vac}}(\boldsymbol{r}, \boldsymbol{r'}, \omega)\delta_{nm} + \boldsymbol{G}^{\mathrm{ref}}_{mn}(\boldsymbol{r}, \boldsymbol{r'}, \omega). \tag{11}$$

Here $\boldsymbol{G}^{\mathrm{vac}}$ is the free dipole field contribution and $\boldsymbol{G}^{\mathrm{ref}}$ is the reflected field contribution to the DGF.

Due to the spherical symmetry of the problem, the DGF of the system is most efficiently expanded onto vector spherical harmonics. The nine different components of the reflection contribution can be worked out by invoking the electromagnetic boundary conditions at the material interfaces. In this work, we consider the situation where the emitter is placed in the inner region of the cavity, and it is therefore only necessary to consider the $n = m = 3$ component of the DGF. We can expand the reflection part of the DGF inside the cavity in terms of vector spherical harmonics,[8,10]

$$\begin{aligned}\boldsymbol{G}^{\mathrm{ref}}_{33}(\boldsymbol{r}, \boldsymbol{r'}, \omega) =& \frac{ik_3}{4\pi}\sum_{e,o}\sum_{n=1}^{\infty}\sum_{m=0}^{n}\frac{(2n+1)(n-m)!}{n(n+1)(n+m)!}(2-\delta_{0,m})\times \\ & \left[r_n^{\mathrm{TE}}(\omega)\boldsymbol{M}^{e}_{o,nm}(\boldsymbol{r}, k_3)\otimes\boldsymbol{M}^{e}_{o,nm}(\boldsymbol{r'}, k_3) + r_n^{\mathrm{TM}}(\omega)\boldsymbol{N}^{e}_{o,nm}(\boldsymbol{r}, k_3)\otimes\boldsymbol{N}^{e}_{o,nm}(\boldsymbol{r'}, k_3)\right].\end{aligned} \tag{12}$$

$k_3 = \omega/c$ because we assume that the inner region is vacuum. $\boldsymbol{M}^{e}_{o,nm}(\boldsymbol{r})$ and $\boldsymbol{N}^{e}_{o,nm}(\boldsymbol{r})$ are

vector spherical harmonics corresponding the transverse electric- and transverse magnetic type modes respectively, and the coefficients $r_n^{\mathrm{TE}}(\omega)$, $r_n^{\mathrm{TM}}(\omega)$ are the reflection coefficients associated with those modes at the interface between the inner cavity region and the cavity shell. The exact expressions for $\boldsymbol{M}^e_{o,nm}(\boldsymbol{r})$, $\boldsymbol{N}^e_{o,nm}(\boldsymbol{r})$, $r_n^{\mathrm{TE}}(\omega)$, and $r_n^{\mathrm{TM}}(\omega)$ have been discussed extensively in the literature and can be found in e.g. Ref.[10]

For a general emitter position inside the cavity, it is necessary to carefully converge the number of vector spherical harmonics used in the calculation of the DGF. In this work, we consider the case where the emitter is placed in the center of the spherical cavity. Dung *et. al.* made the important general observation about the vector spherical harmonics that,[7]

$$\boldsymbol{M}^e_{o,nm}(\boldsymbol{r}, k)|_{kr\to 0} \to (kr)^n, \tag{13}$$

$$\boldsymbol{N}^e_{o,nm}(\boldsymbol{r}, k)|_{kr\to 0} \to (kr)^{n-1}. \tag{14}$$

In the center of the spherical cavity, only the $n = 1$ transverse magnetic mode is therefore of importance. The reflection DGF can consequently be written as,

$$\boldsymbol{G}^{\mathrm{ref}}_{33}(\boldsymbol{r}, \boldsymbol{r'}, \omega)|_{r,r'\to 0} = \frac{i\omega}{6\pi c} r^{\mathrm{TM}}_{n=1}(\omega)\mathbb{1}, \tag{15}$$

where $r^{\mathrm{TM}}_{n=1}(\omega)$ is the reflection coefficient for the lowest order transverse magnetic mode. If we assume that region 2 is very thick we can write,[7]

$$r^{\mathrm{TM}}_{n=1}(\omega) = \frac{\left[i + \rho(n(\omega)+1) - i\rho^2 n(\omega) - \frac{\rho^3 n(\omega)^2}{n(\omega)+1}\right] e^{i\rho}}{\sin\rho - \rho(\cos\rho + in(\omega)\sin\rho) + i\rho^2 n(\omega)\cos\rho - \rho^3(\cos\rho - in(\omega)\sin\rho)\frac{n(\omega)^2}{n(\omega)^2-1}}, \tag{16}$$

where $\rho = R\omega/c$ is the standard size parameter from Mie theory.[9]

The contribution from the vacuum DGF is $\mathrm{Im}\boldsymbol{G}^{\mathrm{vac}}(\boldsymbol{r}, \boldsymbol{r}, \omega) = \frac{\omega}{6\pi c}\mathbb{1}$. The imaginary part

of the full DGF, evaluated in the center of the cavity, thus becomes,

$$\mathrm{Im}\boldsymbol{G}(\mathbf{0},\mathbf{0},\omega)=\frac{\omega}{6\pi c}\left[1+\mathrm{Re}r^{\mathrm{TM}}_{n=1}(\omega)\right]\mathbb{1}. \tag{17}$$

Denoting the unit vector in the i'th direction $\boldsymbol{n}_i$ it can be noticed that, $\boldsymbol{n}_i\cdot\mathrm{Im}\boldsymbol{G}(\mathbf{0},\mathbf{0},\omega)\cdot\boldsymbol{n}_j\propto\delta_{ij}$ which means that the mode orthogonalization needed in the emitter centered formulation is trivial in this case. The cavity field parameters can therefore be derived directly,

$$\boldsymbol{\lambda}_i(\omega)=e\left(\frac{\omega^2}{3\pi^2\epsilon_0 c^3}\left[1+\mathrm{Re}r^{\mathrm{TM}}_{n=1}(\omega)\right]\right)^{1/2}\boldsymbol{n}_i. \tag{18}$$

Notice that the spherical symmetry of the cavity means that at each frequency there are three orthogonal modes with identical field strengths, differing only by their spatial orientation i. Any dipole orientation therefore couples identically to the cavity modes if the emitter is placed in the center of the cavity.

Supplementary Note C: Discretization of the cavity coupling parameters

In practice, we need to discretize the continuous expressions for the cavity field strengths, $\boldsymbol{\lambda}_i(\omega)$. The idea is then to sample densely enough to mimic a true continuum of modes. This means that the sampling density should be converged in the simulations. If we assume that we have uniformly spaced modes we can perform simple discretization,

$$\int_0^\infty d\omega\rightarrow\sum_k\Delta\omega. \tag{19}$$

In the continuous formulation, the operators $\hat{C}_i(\omega)$ have units of $\mathrm{s}^{1/2}$ and the field, $\boldsymbol{E}(\boldsymbol{r},\omega)$ has units of $\mathrm{Vs}^{1/2}/\mathrm{m}$. It therefore makes sense to define a new discretized field and creat-

ing/annihilation operators as,

$$\boldsymbol{E}_{i,k}(\omega_k) \equiv \sqrt{\Delta\omega}\boldsymbol{E}_i(\mathbf{0},\omega_k), \tag{20}$$

$$\hat{a}_{i,k}^{(\dagger)} \equiv \sqrt{\Delta\omega}\hat{C}_i^{(\dagger)}(\omega_k). \tag{21}$$

This also means that we should write,

$$\boldsymbol{\lambda}_{i,k} = e\left(\frac{\Delta\omega\omega_k^2}{3\pi^2\epsilon_0 c^3}\left[1+\operatorname{Re} r_{n=1}^{\mathrm{TM}}(\omega_k)\right]\right)^{1/2}\hat{n}_i. \tag{22}$$

In the discretized version, the number of photon modes will therefore be $N_{\mathrm{modes}} = \mathcal{N} \cdot N_k$, where $\mathcal{N}$ is the number of bright modes and N_k is the number of frequency points used in the discretization. As mentioned in the main text, in our case this number is 3 and the sums in the discretized version therefore includes $N_{\mathrm{modes}} = 3N_k$ photon modes. We will use the composite index $\alpha = (i,k)$ to refer to this set of discrete modes and this is thus the α which appears in the generalized Casida formulation discussed in Section 2.2 of the main manuscript.

Supplementary Note D: Computational details for the QEDFT calculations

We use the publicly available real space psuedo-potential DFT code OCTOPUS.[11] Molecular geometries are optimized with the LDA exchange-correlation functional, on a real space grid consisting of spheres of radius 6Å around each atom with a grid spacing of 0.16Å.

We also calculate the ground state using the LDA exchange-correlation functional on a real space grid consisting of spheres of radius 6 Å around each atom with a grid spacing of 0.08 Å. We perform a fixed-density calculation on top of the ground state calculation to determine the excited states needed for the Casida calculation for which we use 500 excited

states. These parameters ensure that the relevant transitions are converged to within 1 meV/atom.

We employ uniform sampling of the electromagnetic environment and converge each calculation separately. In general, we find good convergence when sampling each cavity peak with around 1000 photon modes.

Supplementary Note E: Connection to the standard light-matter coupling strengths used in quantum optics

In quantum optics, the description of the electronic system is commonly restricted to a few electronic states. It is therefore standard to represent the light-matter interaction Hamiltonian in terms of the light-matter coupling strength g instead of cavity field strengths $\boldsymbol{\lambda}$. For a transition between two electronic states $|e_1\rangle$ and $|e_2\rangle$, the light-matter coupling strength is $g = \hbar^{-1}\boldsymbol{d} \cdot \boldsymbol{E}(\boldsymbol{r}_0)$ where $\boldsymbol{d} = -e\langle e_1|\hat{\boldsymbol{R}}|e_2\rangle$ is the transition dipole matrix element of the transition of interest and $\boldsymbol{E}(\boldsymbol{r}_0)$ is the electric field matrix element. Importantly, unlike the cavity field strength parameters which only relates to the cavity geometry, g is a direct measure of the light-matter coupling strength for a given electronic transition. It is thus a property of both light and matter specific to the situation under consideration.

To directly establish the connection between the light-matter coupling strength and the cavity field parameters, we note that for a single electronic transition as described above, the light-matter interaction Hamiltonian in terms of the bright modes can be written as,

$$\hat{H}_{\text{int}} = -\hat{\boldsymbol{d}} \cdot \hat{\boldsymbol{E}}(\boldsymbol{r}_0) = \sum_i \int_0^\infty \hbar g_i(\omega) \left(\hat{C}_i(\omega) + \hat{C}_i^\dagger(\omega)\right) \left(\hat{\sigma}^+ + \hat{\sigma}^-\right) \tag{23}$$

where $\hat{\sigma}^+ = |e_2\rangle\langle e_1|$, $\hat{\sigma}^- = |e_1\rangle\langle e_2|$ are the transition operators for the electronic transition and $\hat{C}_i^{(\dagger)}(\omega)$ are the creation(annihilation) operators of the bright modes of the electromagnetic field. Finally, $g_i(\omega) \equiv \hbar^{-1}\boldsymbol{d} \cdot \boldsymbol{E}_i(\boldsymbol{r}_0, \omega)$ is the light-matter coupling strength. Recalling

that $\boldsymbol{\lambda}_i(\omega) = \left(\frac{2}{\hbar\omega}\right)^{1/2} e\boldsymbol{E}_i(\boldsymbol{r}_0, \omega)$ we can state that the light-matter coupling strength corresponding to a given cavity field strength and electronic dipole moment is,

$$\hbar g_i(\omega) = \left(\frac{\hbar\omega}{2e^2}\right)^{1/2} \boldsymbol{d} \cdot \boldsymbol{\lambda}_i(\omega). \tag{24}$$

Light-matter coupling strengths for benzene in the spherical cavity:

For the spherical cavity the modes are uniformly polarized and we may therefore write,

$$g_i(\omega) = \left(\frac{\hbar\omega}{2e^2}\right)^{1/2} |\boldsymbol{d}||\boldsymbol{\lambda}_i(\omega)| \tag{25}$$

The total coupling strength of a given cavity mode peak is thus,

$$g_{\text{eff}} \equiv \int_p d\omega \left(\frac{\hbar\omega}{2e^2}\right)^{1/2} |\boldsymbol{d}||\boldsymbol{\lambda}_i(\omega)| \approx \left(\frac{\hbar\omega_c}{2e^2}\right)^{1/2} |\boldsymbol{d}|\lambda_c \tag{26}$$

where ω_c is the center frequency of the cavity peak, and the p subscript denotes an integration across one peak in the spectral density of the spherical cavity as in Eq. 23 of the main manuscript. From the Casida calculation we can extract the in-plane dipole moment of the $\Pi \rightarrow \Pi^*$ transition in benzene which comes out to $0.096\, e \cdot \text{nm}$. Using the λ_c from Fig. 2 in the manuscript we find $\hbar g_{\text{eff}} = 3.81$ meV, $\hbar g_{\text{eff}} = 0.62$ meV and $\hbar g_{\text{eff}} = 0.20$ meV for $R = 16$ nm, $R = 149$nm and $R = 1704$ nm respectively. Again we emphasize that these coupling strengths are a property of *both* the electromagnetic environment and the specific transition in the benzene molecule. One should therefore be careful with general conclusions based on this number.

Supplementary Note F: The Fabry Perot cavity

In this supplementary note we briefly discuss the widely used Fabry Perot cavity (FPC). As shown in Supplementary Figure 1a, the stereotypical FPC is a stratified system. Con-

sequently, its DGF can be conveniently represented in the angular spectrum representation. Considering just the cavity region, the DGF can be written as,[8]

$$\boldsymbol{G}(\boldsymbol{r},\boldsymbol{r}',\omega)=\frac{i}{8\pi^2}\int\frac{d\boldsymbol{q}}{k_z q^2}\left[\boldsymbol{M}(\boldsymbol{q},\boldsymbol{r},\boldsymbol{r'})+\boldsymbol{N}(\boldsymbol{q},\boldsymbol{r},\boldsymbol{r'})\right]. \tag{27}$$

Here $\boldsymbol{q}$ is the in-plane wave vector, $k_z=\sqrt{k^2-q^2}$ is the out of plane wave vector where k is the wave number in the medium and $q=|\boldsymbol{q}|$. $\boldsymbol{M}$ and $\boldsymbol{N}$ are the tensorial contributions from the TE and TM polarized modes respectively. These tensors can be found by solving the scattering from a point source in a layered medium as discussed in detail in e.g. Refs.[8,9] Importantly, if we consider a single emitter position in the FPC, we only need information about the $\boldsymbol{r}=\boldsymbol{r'}$ part of the DGF to describe the coupling to the electromagnetic environment. Conveniently, at $\boldsymbol{r}=\boldsymbol{r'}$ both $\boldsymbol{M}$ and $\boldsymbol{N}$ are diagonal matrices when expressed in cylindrical coordinates with the z-axis perpendicular to the mirrors,

$$k_x=q\cos\phi,\qquad k_y=q\sin\phi,\qquad k_z=k_z. \tag{28}$$

This is also the natural choice because the in-plane wave number $q=\sqrt{k_x^2+k_y^2}$ is the natural variable in the stratified FPC. The integration over ϕ can be performed analytically which results in,

$$\boldsymbol{G}^{\mathrm{TE}}(\boldsymbol{r},\boldsymbol{r},\omega)=\int dqq\int_0^{2\pi}d\phi\,\boldsymbol{M}(\boldsymbol{q},\boldsymbol{r},\boldsymbol{r})=\frac{i}{8\pi}\int dq\frac{q}{k_z}\begin{bmatrix}1&0&0\\0&1&0\\0&0&0\end{bmatrix}R_{\|}^{\mathrm{TE}}(z), \tag{29}$$

and,

$$\boldsymbol{G}^{\mathrm{TM}}(\boldsymbol{r},\boldsymbol{r},\omega) =$$

$$\int dqq \int_0^{2\pi} d\phi\, \boldsymbol{N}(\boldsymbol{q},\boldsymbol{r},\boldsymbol{r}) = \frac{i}{8\pi k^2}\int dq \begin{bmatrix} qk_z R_{\parallel}^{\mathrm{TM}}(z) & 0 & 0 \\ 0 & qk_z R_{\parallel}^{\mathrm{TM}}(z) & 0 \\ 0 & 0 & 2q^3/k_z R_{\perp}^{\mathrm{TM}}(z). \end{bmatrix} \tag{30}$$

Taking the emitter to be placed at $z = 0$ and denoting the distance between the emitter and the top and bottom mirrors as t and b respectively, the reflection functions can be written as,

$$R_{\perp}^{\mathrm{TM}}(z,q) = \frac{(1 + r_{cb}^{\mathrm{TM}} e^{2ik_z b})(1 + r_{ct}^{\mathrm{TM}} e^{2ik_z t})}{1 - r_{cb}^{\mathrm{TM}} r_{ct}^{\mathrm{TM}} e^{2ik_z d}} \tag{31}$$

$$R_{\parallel}^{\mathrm{TE}}(z,q) = \frac{(1 + r_{cb}^{\mathrm{TE}} e^{2ik_z b})(1 + r_{ct}^{\mathrm{TE}} e^{2ik_z t})}{1 - r_{cb}^{\mathrm{TE}} r_{ct}^{\mathrm{TE}} e^{2ik_z d}} \tag{32}$$

$$R_{\parallel}^{\mathrm{TM}}(z,q) = \frac{(1 - r_{cb}^{\mathrm{TM}} e^{2ik_z b})(1 - r_{ct}^{\mathrm{TM}} e^{2ik_z t})}{1 - r_{cb}^{\mathrm{TM}} r_{ct}^{\mathrm{TM}} e^{2ik_z d}} \tag{33}$$

The subscripts cb and ct refers to the interfaces between the cavity region and the bottom- and top mirror respectively. We use the following definition of the Fresnel coefficients for light incident on the interface between two regions a and b from region a,

$$r_{ab}^{\mathrm{TE}} = \frac{k_z^a - k_z^b}{k_z^a + k_z^b}, \tag{34}$$

$$r_{ab}^{\mathrm{TM}} = \frac{\epsilon_b k_z^a - \epsilon_a k_z^b}{\epsilon_b k_z^a + \epsilon_a k_z^b}. \tag{35}$$

Note that these Fresnel coefficients could be replaced with the generalized Fresnel coefficients if more complicated mirrors such as e.g. distributed Bragg reflectors were considered.

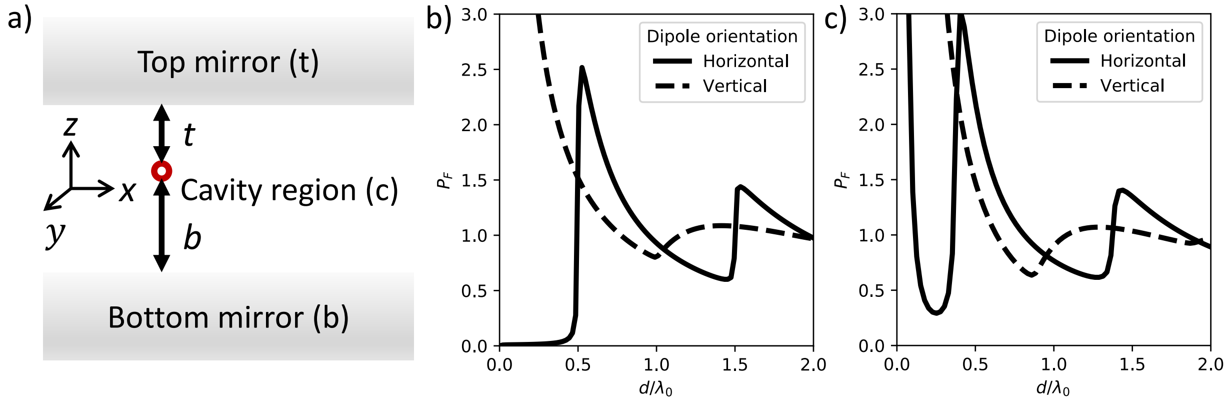

Supplementary Figure 1: **The Fabry Perot cavity:** a) Paradigmatic sketch of a Fabry Perot Cavity. b) Purcell enhancement as a function of d/λ_0 for both vertical and horizontal dipole orientations when considering idealized mirrors with a frequency independent reflectivity $R = 0.95$. c) Purcell enhancement as a function of d/λ_0 for both vertical and horizontal dipole orientations when considering gold mirrors with dielectric functions described by the Drude model in Eq. 23 of the main manuscript.

Light-matter interaction in the Fabry-Perot cavity

Because the DGF is diagonal at $\boldsymbol{r} = \boldsymbol{r'}$, there is no coupling between the horizontal and vertical dipole orientations by feedback from the electromagnetic environment. Consequently, the local density of states (LDOS),

$$\rho(\boldsymbol{r},\omega) = \frac{6\omega}{\pi c^2}\hat{n}_D \cdot \mathrm{Im}\left[\boldsymbol{G}(\boldsymbol{r},\boldsymbol{r},\omega)\right] \cdot \hat{n}_D. \tag{36}$$

for either of these two dipole orientations, denoted by $\hat{n}_D$, becomes a direct proxy for the light-matter coupling strength. It can therefore be used evaluate the potential of FPCs for realizing single/few emitter strong coupling. One concrete measure of the FPCs alterations of the electromagnetic environment is its modifications of the free space density of states. This is measured directly by the Purcell enhancement,

$$P_F(\boldsymbol{r},\omega) = \frac{\rho(\boldsymbol{r},\omega)}{\rho_0(\omega)}, \tag{37}$$

which is given as a rescaling of the LDOS by the free space density of states of the electromagnetic field, $\rho_0(\omega)$. To explore this, we consider two different FPCs, both of which consist of a central cavity region made of vacuum, surrounded by mirrors. In one instance, we consider idealized mirrors with a constant reflectivity of 0.95, and in the other we consider mirrors made of a Drude metal. In the latter case, we use the Drude parameters for gold[12] as also done in the main manuscript and consider frequencies below the plasma frequency such that the mirrors retain their reflectivity. Figure 1(b,c) shows the Purcell enhancement as a function of mirror distance, d, for a fixed emitter wavelength, λ_0, for idealized mirrors (b) and for Drude mirrors (c). In both cases, the horizontal and vertical dipole orientations are considered separately because, as mentioned above, the cavity does not couple the two.

Starting with Figure 1b for the horizontal dipole orientation, we observe only relatively broad cavity resonances with modest Purcell enhancements of maximum ~ 2.5. This reflects the relatively weak concentration of the electromagnetic field in the FPC and emphasizes that the case with idealized mirrors is ill-suited for single point emitter strong coupling. We emphasize that the widths of these resonances are only weakly linked to the finite mirror reflectivity, and instead reflect the fact that the cavity features dispersion in plane. As such, these peaks would retain a finite width even in the limit of perfect mirror reflectivity.[13] For $d < \lambda_0/2$ we observe a near complete suppression of the coupling of the horizontal dipole orientation to the electromagnetic environment. This happens because the horizontal dipole always couples to the $q = 0$ mode. This mode will not exist for $d < \lambda_0/2$ with perfect mirrors and be strongly suppresses for near-perfect mirrors. This suppression can also be understood as a result of the mirror charge effect which will result in emission suppression for emitters with horizontal transition dipole moments when these are placed close to idealized reflective surfaces.[1] For the vertical dipole orientation, we generally observe relatively weak, near unity Purcell enhancement, except when d becomes smaller than $\sim \lambda_0/2$. From this point, the Purcell enhancement diverges as $d \to 0$. This divergence can be understood as a result of concentration of energy into the mode propagating parallel to the mirrors when the mirror

distance is decreased.[13] Alternatively, it can also be more intuitively understood as a result of the mirror charge effect which enhances emission for vertical dipole orientations. For a single reflective interface, this enhancement would be a simple factor of 2.[1] However, in the double mirror case, the image charge dipole in one mirror is enhanced by its mirror charge dipole in the second mirror and vice versa to infinity. Therefore, the Purcell enhancement diverges as $d \to 0$ instead of approaching a simple factor of 2. When we consider the more realistic situation with mirrors described by the Drude model in Figure 1 c we observe more or less the same as was the case with idealized mirrors. The only major difference is that we observe a divergence for the horizontal dipole orientation at small mirror distances. This happens due to near field coupling to the metal primarily representing quenching. We note that this also happens to the vertical dipole orientation but that this is less visible because of the mirror charge effect already causing a divergent behaviour for $d \to 0$ as was also the case with idealized mirrors.

The above discussion shows that the FPC provides only modest modification of the electromagnetic environment relative to the free space case with broad resonances whose width arise from the in-place dispersion of the cavity. The exception to this statement is for very small mirror spacing. However, in this latter regime the electromagnetic environment does not show resonant behaviour. This is also manifested in the cavity field strengths. In Supplementary Figure 2(a,b), we present the cavity field strengths for two different FPC setups: (a) idealized mirrors with a top mirror reflectivity of 0.95 and unity bottom mirror reflectivity and (b) Drude metal mirrors with a dielectric function described by Eq. 23 in the main manuscript. In both cases, we consider a mirror spacing of 700 nm. As shown in Supplementary Figure 2a, the cavity field strengths are only weakly modified relative to their free space equivalent (depicted by the dashed line) for both horizontal and vertical dipole orientations in the FPC with idealized mirrors, except for very small mirror spacing, as discussed above. For the more realistic Drude mirrors (Supplementary Figure 2b), the

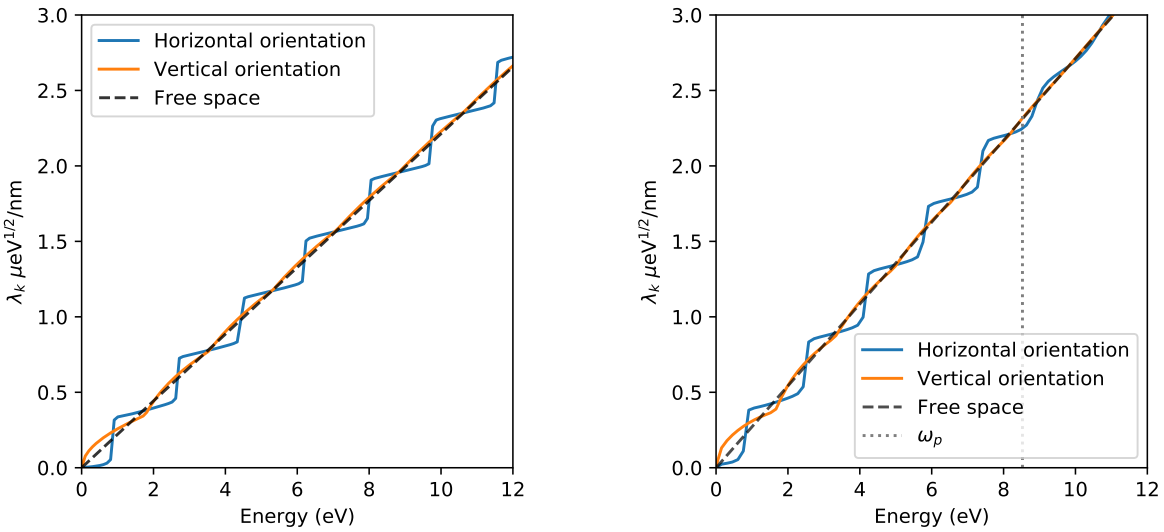

(a) (b)

Supplementary Figure 2: Cavity field parameters in a Fabry Perot cavity with idealized mirrors with a reflectivity of 0.95 (a) and one with mirrors made with gold mirrors with dielectric functions described by the Drude model in Eq. 23 of the main manuscript. In both cases, we consider a mirror separation of 700 nm. The sampling density employed in this figure is 10 points/eV.

results are similar. The plasma frequency of the metallic mirror is marked by the dotted line. Similar to the spherical cavity in the main text, we observe that above the plasma frequency, the mirror loses its reflectivity, leading to the loss of structure in the cavity field strengths. This again emphasizes the direct connection of geometry and material composition with the mode structure of the electromagnetic environment.

For these reasons, we conclude that the FPC is ill-suited for single- and few emitter vacuum strong coupling. However, the FPC can still be suited for collective strong coupling[14] and coupling to extended systems[15] where the extended modes of the electromagnetic environment can be sampled more effectively and in-plane momentum conservation can result in mode selectivity in the light-matter coupling.

References

(1) Scheel, S.; Buhmann, S. Y. Macroscopic QED-concepts and applications. *acta physica slovaca* **2008**, 675–809.

(2) Flick, J.; Welakuh, D. M.; Ruggenthaler, M.; Appel, H.; Rubio, A. Light–matter response in nonrelativistic quantum electrodynamics. *ACS photonics* **2019**, *6*, 2757–2778.

(3) Wang, D. S.; Neuman, T.; Flick, J.; Narang, P. Light–matter interaction of a molecule in a dissipative cavity from first principles. *J. Chem. Phys.* **2021**, *154*, 104109.

(4) Mukamel, S. *Principles of nonlinear optical spectroscopy*; Oxford University Press on Demand, 1999.

(5) Flick, J.; Rivera, N.; Narang, P. Strong light-matter coupling in quantum chemistry and quantum photonics. *Nanophotonics* **2018**, *7*, 1479–1501.

(6) Chen, Y.; Nielsen, T. R.; Gregersen, N.; Lodahl, P.; Mørk, J. Finite-element modeling of spontaneous emission of a quantum emitter at nanoscale proximity to plasmonic waveguides. *Phys. Rev. B* **2010**, *81*, 125431.

(7) Dung, H. T.; Knöll, L.; Welsch, D.-G. Spontaneous decay in the presence of dispersing and absorbing bodies: General theory and application to a spherical cavity. *Phys. Rev. A* **2000**, *62*, 053804.

(8) Chew, W. C. *Waves and fields in inhomogenous media*; John Wiley & Sons, 1999; Vol. 16.

(9) Novotny, L.; Hecht, B. *Principles of nano-optics*; Cambridge university press, 2012.

(10) Li, L.-W.; Kooi, P.-S.; Leong, M.-S.; Yee, T.-S. Electromagnetic dyadic Green's function in spherically multilayered media. *IEEE Transactions on Microwave Theory and Techniques* **1994**, *42*, 2302–2310.

(11) Tancogne-Dejean, N. et al. Octopus, a computational framework for exploring light-driven phenomena and quantum dynamics in extended and finite systems. *J. Chem. Phys.* **2020**, *152*, 124119.

(12) Blaber, M. G.; Arnold, M. D.; Ford, M. J. Search for the ideal plasmonic nanoshell: the effects of surface scattering and alternatives to gold and silver. *J. Phys. Chem. C* **2009**, *113*, 3041–3045.

(13) Dutra, S.; Knight, P. Spontaneous emission in a planar Fabry-Pérot microcavity. *Phys. Rev. A* **1996**, *53*, 3587.

(14) Thomas, A.; Lethuillier-Karl, L.; Nagarajan, K.; Vergauwe, R. M. A.; J. George, T. C.; Shalabney, A.; Devaux, E.; Genet, C.; Moran, J.; Ebbesen, T. W. Tilting a ground-state reactivity landscape by vibrational strong coupling. *Science* **2019**, *364*, 615–619.

(15) Gu, J.; Walther, V.; Waldecker, L.; Rhodes, D.; Raja, A.; Hone, J. C.; Heinz, T. F.; Kéna-Cohen, S.; Pohl, T.; Menon, V. M. Enhanced nonlinear interaction of polaritons via excitonic Rydberg states in monolayer WSe2. *Nat. Comm.* **2021**, *12*, 2269.
